# Josephson effect in junctions of conventional and topological superconductors

**DOI:** 10.3762/bjnano.9.158

**Published:** 2018-06-06

**Authors:** Alex Zazunov, Albert Iks, Miguel Alvarado, Alfredo Levy Yeyati, Reinhold Egger

**Affiliations:** 1 Institut für Theoretische Physik, Heinrich-Heine-Universität, D-40225 Düsseldorf, Germany; 2 Departamento de Física Teórica de la Materia Condensada C-V, Condensed Matter Physics Center (IFIMAC) and Instituto Nicolás Cabrera, Universidad Autónoma de Madrid, E-28049 Madrid, Spain

**Keywords:** Andreev bound states, Josephson current–phase relation, Majorana zero modes, topological superconductivity

## Abstract

We present a theoretical analysis of the equilibrium Josephson current-phase relation in hybrid devices made of conventional *s*-wave spin-singlet superconductors (S) and topological superconductor (TS) wires featuring Majorana end states. Using Green’s function techniques, the topological superconductor is alternatively described by the low-energy continuum limit of a Kitaev chain or by a more microscopic spinful nanowire model. We show that for the simplest S–TS tunnel junction, only the *s*-wave pairing correlations in a spinful TS nanowire model can generate a Josephson effect. The critical current is much smaller in the topological regime and exhibits a kink-like dependence on the Zeeman field along the wire. When a correlated quantum dot (QD) in the magnetic regime is present in the junction region, however, the Josephson current becomes finite also in the deep topological phase as shown for the cotunneling regime and by a mean-field analysis. Remarkably, we find that the S–QD–TS setup can support φ_0_-junction behavior, where a finite supercurrent flows at vanishing phase difference. Finally, we also address a multi-terminal S–TS–S geometry, where the TS wire acts as tunable parity switch on the Andreev bound states in a superconducting atomic contact.

## Introduction

The physics of topological superconductors (TSs) is being vigorously explored at present. After Kitaev [1] showed that a one-dimensional (1D) spinless fermionic lattice model with nearest-neighbor *p*-wave pairing (‘Kitaev chain’) features a topologically nontrivial phase with Majorana bound states (MBSs) at open boundaries, references [2–3] have pointed out that the physics of the Kitaev chain could be realized in spin–orbit coupled nanowires with a magnetic Zeeman field and in the proximity to a nearby *s*-wave superconductor. The spinful nanowire model of references [2–3] indeed features *p*-wave pairing correlations for appropriately chosen model parameters. In addition, it also contains *s*-wave pairing correlations which become gradually smaller as one moves into the deep topological regime. Topologically nontrivial hybrid semiconductor nanowire devices are of considerable interest in the context of quantum information processing [4–12], and they may also be designed in two-dimensional layouts by means of gate lithography techniques. Over the last few years, several experiments employing such platforms have provided mounting evidence for MBSs, e.g., from zero-bias conductance peaks in N–TS junctions (where N stands for a normal-conducting lead) and via signatures of the 4π-periodic Josephson effect in TS–TS junctions [13–25]. Related MBS phenomena have been reported for other material platforms as well [26–30], and most of the results reported below also apply to those settings. Available materials are often of sufficiently high quality to meet the conditions for ballistic transport, and we will therefore neglect disorder effects.

In view of the large amount of published theoretical works on the Josephson effect in such systems, let us first motivate the present study. (For a more detailed discussion and references, see below.) Our manuscript addresses the supercurrent flowing in Josephson junctions with a magnetic impurity. By considering Josephson junctions between a topological superconductor and a non-topological superconductor, we naturally extend previous works on Josephson junctions with a magnetic impurity between two conventional superconductors, as well as other works on Josephson junctions between topological and non-topological superconductors but without a magnetic impurity. In the simplest description, Josephson junctions between topological and non-topological supeconductors carry no supercurrent. Instead, a supercurrent can flow only with certain deviations from the idealized model description. The presence of a magnetic impurity in the junction is one of these deviations, and this effect allows for novel signatures for the topological transition via the so-called φ_0_-behavior and/or through the kink-like dependence of the critical current on a Zeeman field driving the transition. We consider two different geometries in various regimes, e.g., the cotunneling regime where a controlled perturbation theory is possible, and a mean-field description of the stronger-coupling regime. We study both idealized Hamiltonians (allowing for analytical progress) as well as more realistic models for the superconductors.

**Figure 1:**
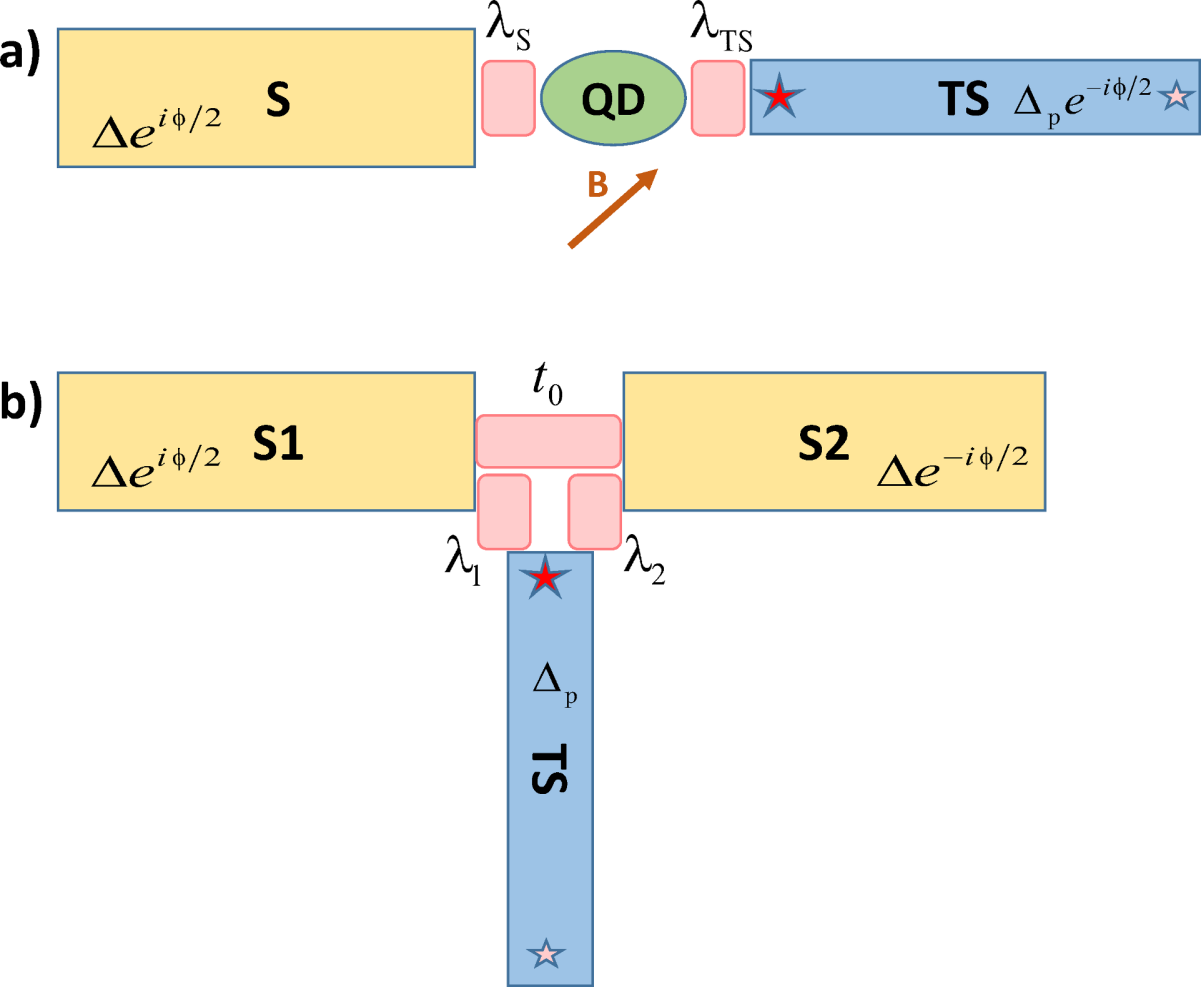
Schematic setups studied in this paper. a) S–QD–TS geometry: S denotes a conventional *s*-wave BCS superconductor with order parameter 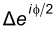, and TS represents a topologically nontrivial superconducting wire with MBSs (shown as stars) and proximity-induced order parameter 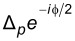. The interface contains a quantum dot (QD) corresponding to an Anderson impurity, connected to the S/TS leads by tunnel amplitudes λ_S/TS_ (light red). The QD is also exposed to a local Zeeman field **B**. b) S–TS–S geometry: Two conventional superconductors (S1 and S2) with the same gap Δ and a TS wire with proximity gap Δ_p_ form a trijunction. The order parameter phase of S1 (S2), 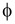
_1_ = 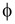/2 (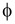
_2_ = −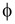/2), is taken relative to the phase of the TS wire, and tunnel couplings λ_1/2_ connect S1/S2 to the TS wire. When the TS wire is decoupled (λ_1,2_ = 0), the S–S junction becomes a standard SAC with transparency 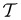 determined by the tunnel amplitude *t*_0_, see Equation 1.

To be more specific, we address the equilibrium current–phase relation (CPR) in different setups involving both conventional *s*-wave BCS superconductors (‘S’ leads) and TS wires, see Figure 1 for a schematic illustration. In general, the CPR is closely related to the Andreev bound state (ABS) spectrum of the system. For S–TS junctions with the TS wire deep in the topological phase such that it can be modeled by a Kitaev chain, the supercurrent vanishes identically [31]. This supercurrent blockade can be traced back to the different (*s*/*p*-wave) pairing symmetries for the S/TS leads, together with the fact that MBSs have a definite spin polarization. For an early study of Josephson currents between superconductors with different (*p*/*d*) pairing symmetries, see also [32]. A related phenomenon concerns Multiple Andreev Reflection (MAR) features in nonequilibrium superconducting quantum transport at subgap voltages [33–36]. Indeed, it has been established that MAR processes are absent in S–TS junctions (with the TS wire in the deep topological regime) such that only quasiparticle transport above the gap is possible [37–44].

There are several ways to circumvent this supercurrent blockade in S–TS junctions. (i) One possibility has been described in [43]. For a trijunction formed by two TS wires and one S lead, crossed Andreev reflections allow for the nonlocal splitting of Cooper pairs in the S electrode involving both TS wires (or the reverse process). In this way, an equilibrium supercurrent will be generated unless the MBS spin polarization axes of both TS wires are precisely aligned. (ii) Even for a simple S–TS junction, a finite Josephson current is expected when the TS wire is modeled as spinful nanowire. This effect is due to the residual *s*-wave pairing character of the spinful TS model [2–3]. Interestingly, upon changing a control parameter, e.g., the bulk Zeeman field, which drives the TS wire across the topological phase transition, we find that the critical current exhibits a kink-like feature that is mainly caused by a suppression of the Andreev state contribution in the topological phase. (iii) Yet another possibility is offered by junctions containing a magnetic impurity in a local magnetic field. We here analyze the S–QD–TS setup in Figure 1a in some detail, where a quantum dot (QD) is present within the S–TS junction region. The QD is modeled as an Anderson impurity [36], which is equivalent to a spin-1/2 quantum impurity over a wide parameter regime. Once spin mixing is induced by the magnetic impurity and the local magnetic field, we predict that a finite Josephson current flows even in the deep topological limit. In particular, in the cotunneling regime, we find an anomalous Josephson effect with finite supercurrent at vanishing phase difference (φ_0_-junction behavior) [45–47], see also [48–51]. The 2π-periodic CPR found in S–QD–TS junctions could thereby provide independent evidence for MBSs via the anomalous Josephson effect. In addition, we compute the CPR within the mean-field approximation in order to go beyond perturbation theory in the tunnel couplings connecting the QD to the superconducting leads. Our mean-field analysis shows that the φ_0_-junction behavior is a generic feature for S–QD–TS devices in the topological regime which is not limited to the cotunneling regime.

In the final part of the paper, we turn to the three-terminal S–TS–S setup shown in Figure 1b, where the S–S junction by itself (with the TS wire decoupled) represents a standard superconducting atomic contact (SAC) with variable transparency of the weak link. Recent experiments have demonstrated that the many-body ABS configurations of a SAC can be probed and manipulated to high accuracy by microwave spectroscopy [52–54]. When the TS wire is coupled to the S–S junction, see Figure 1b, the Majorana end state acts as a parity switch on the ABS system of the SAC. This effect allows for additional functionalities in Andreev spectroscopy. We note that similar ideas have also been explored for TS–N–TS systems [55].

## Results and Discussion

### S–QD–TS junction

#### Model

Let us start with the case of an S–QD–TS junction, where an interacting spin-degenerate single-level quantum dot (QD) is sandwiched between a conventional *s*-wave superconductor (S) and a topological superconductor (TS). This geometry is shown in Figure 1a. The corresponding topologically trivial S–QD–S problem has been studied in great detail over the past decades both theoretically [56–63] and experimentally [64–69]. A main motivation for those studies came from the fact that the QD can be driven into the magnetic regime where it represents a spin-1/2 impurity subject to Kondo screening by the leads. The Kondo effect then competes against the superconducting bulk gap and one encounters local quantum phase transitions. By now, good agreement between experiment and theory has been established. Rather than studying the fate of the Kondo effect in the S–QD–TS setting of Figure 1a, we here pursue two more modest goals. First, we shall discuss the cotunneling regime in detail, where one can employ perturbation theory in the dot–lead couplings. This regime exhibits π-junction behavior in the S–QD–S case [56]. Second, in order to go beyond the cotunneling regime, we have performed a mean-field analysis similar in spirit to earlier work for S–QD–S devices [57–58].

The Hamiltonian for the setup in Figure 1a is given by

[2]
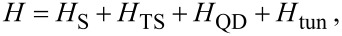


where *H*_S/TS_ and *H*_QD_ describe the semi-infinite S/TS leads and the isolated dot in between, respectively, and *H*_tun_ refers to the tunnel contacts. We often use units with *e* = 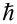 = *k*_B_ = 1, and β = 1/*T* denotes inverse temperature. The QD is modeled as an Anderson impurity [36], i.e., a single spin-degenerate level of energy ε_0_ with repulsive on-site interaction energy *U >* 0,

[3]
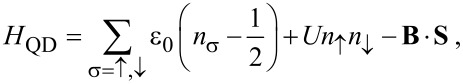


where the QD occupation numbers are *n*_σ_ = 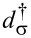 *d*_σ_ = 0,1, with dot fermion operators *d*_σ_ and 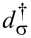 for spin σ. Using standard Pauli matrices σ*_x,y,z_*, we define

[4]
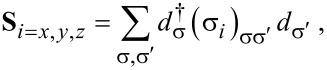


such that **S**/2 is a spin-1/2 operator. In the setup of Figure 1a, we also take into account an external Zeeman field **B** = (*B_x_*, *B_y_*, *B_z_*) acting on the QD spin, where the units in Equation 3 include gyromagnetic and Bohr magneton factors. The spinful nanowire proposal for TS wires [2–3] also requires a sufficiently strong bulk Zeeman field oriented along the wire in order to realize the topologically nontrivial phase, but for concreteness, we here imagine the field **B** as independent local field coupled only to the QD spin. One could use, e.g., a ferromagnetic grain near the QD to generate it. This field here plays a crucial role because for **B** = 0, the S+QD part is spin rotation [SU(2)] invariant and the arguments of [31] then rule out a supercurrent for TS wires in the deep topological regime. We show below that unless **B** is inadvertently aligned with the MBS spin polarization axis, spin mixing will indeed generate a supercurrent.

The S/TS leads are coupled to the QD via a tunneling Hamiltonian [70],

[5]
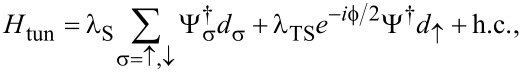


where ψ_σ_ and ψ are boundary fermion fields representing the S lead and the effectively spinless TS lead, respectively. For the S lead, we assume the usual BCS model [62], where the operator ψ_σ_ annihilates an electron with spin σ at the junction. The TS wire will, for the moment, be described by the low-energy Hamiltonian of a Kitaev chain in the deep topological phase with chemical potential μ = 0 [1,5]. The corresponding fermion operator ψ at the junction includes both the MBS contribution and above-gap quasiparticles [40]. Without loss of generality, we choose the unit vector 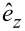 as the MBS spin polarization direction and take real-valued tunnel amplitudes λ_S/TS_, see Figure 1a, using a gauge where the superconducting phase difference 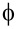 appears via the QD–TS tunneling term. These tunnel amplitudes contain density-of-states factors for the respective leads. The operator expression for the current flowing through the system is then given by

[6]
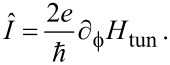


We do not specify *H*_S/TS_ in Equation 2 explicitly since within the imaginary-time (τ) boundary Green’s function (bGF) formalism [40] employed here, we only need to know the bGFs. For the S lead with gap value Δ, the bGF has the Nambu matrix form [40]

[7]
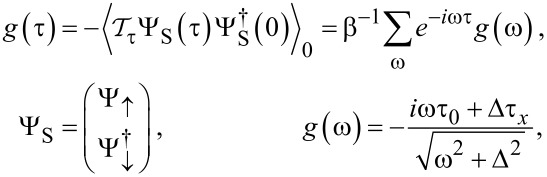


where the expectation value 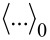 refers to an isolated S lead, 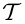 denotes time ordering, ω runs over fermionic Matsubara frequencies, i.e., ω = 2π(*n* + 1/2)/β with integer *n*, and we define Pauli (unity) matrices τ*_x,y,z_* (τ_0_) in particle–hole space corresponding to the Nambu spinor Ψ_S_. Similarly, for a TS lead with proximity-induced gap Δ_p_, the low-energy limit of a Kitaev chain yields the bGF [40]

[8]
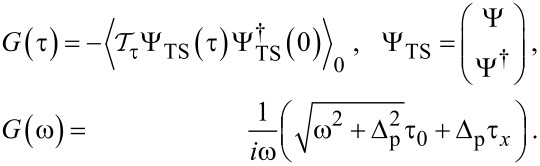


The matrices τ_0_*_,x_* here act in the Nambu space defined by the spinor Ψ_TS_. Later on we will address how our results change when the TS wire is modeled as spinful nanowire [2–3], where the corresponding bGF has been specified in [43]. We emphasize that the bGF (Equation 8) captures the effects of both the MBS (via the 1/ω term) and of the above-gap continuum quasiparticles (via the square root) [40,71].

In most of the following discussion, we will assume that *U* is the dominant energy scale, with the single-particle level located at ε_0_ ≈ − *U*/2. In that case, low-energy states with energy well below *U* are restricted to the single occupancy sector,

[9]
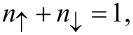


and the QD degrees of freedom become equivalent to the spin-1/2 operator **S**/2 in Equation 4. In this regime, the QD acts like a magnetic impurity embedded in the S–TS junction. Using a Schrieffer–Wolff transformation to project the full Hamiltonian to the Hilbert subspace satisfying Equation 9, *H* → *H*_eff_, one arrives at the effective low-energy Hamiltonian

[10]
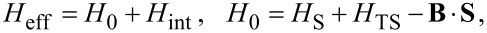


with the interaction term

[11]
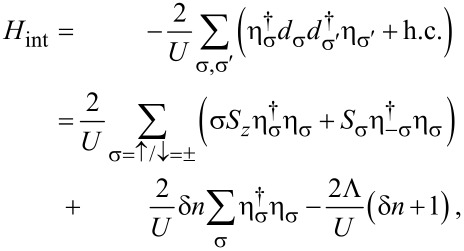


where *S*_±_ = *S_x_* ± *iS_y_* and δ*n* = 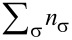 − 1. Moreover, 
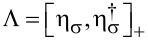
 is the anticommutator of the composite boundary fields

[12]
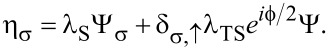


We note that Λ is real-valued and does not depend on 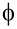. Due to the constraint (Equation 9) on the dot occupation, the last two terms in Equation 11 do not contribute to the system dynamics and we obtain

[13]
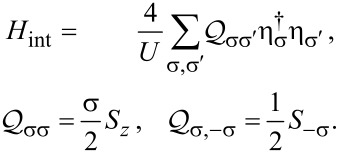


A formally exact expression for the partition function is then given by

[14]
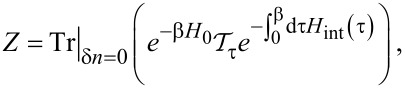


where 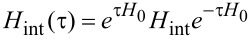 with 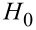 in Equation 10 and the trace extends only over the Hilbert subspace corresponding to Equation 9. We can equivalently write Equation 14 in the form

[15]
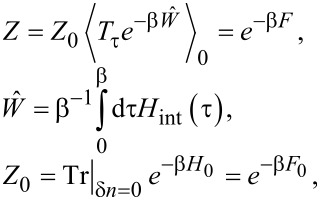


where *F* is the free energy. The Josephson current then follows as *I* =(2*e*/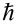) ∂
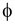
*F*, see Equation 6.

#### Cotunneling regime

We now address the CPR in the elastic cotunneling regime,

[16]
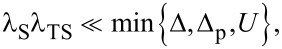


where perturbation theory in *H*_int_ is justified. We thus wish to compute the free energy *F*(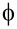) from Equation 15 to lowest nontrivial order. With *W*_0_ = 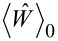, the standard cumulant expansion gives

[17]
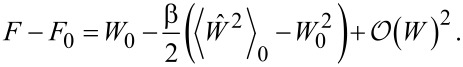


By virtue of Wick’s theorem, time-ordered correlation functions of the boundary operators (Equation 12) are now expressed in terms of S/TS bGF matrix elements, see Equation 7 and Equation 8,

[18]
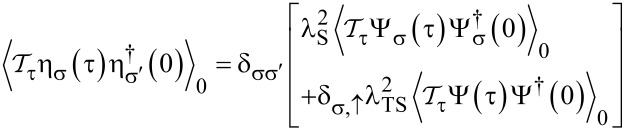


and similarly

[19]
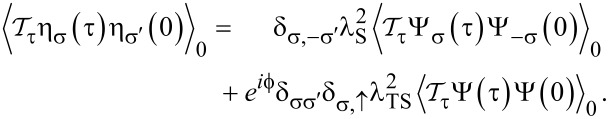


Next we observe that 
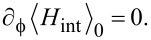
 As a consequence, the 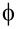-independent terms *W*_0_ and 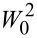 in Equation 17 do not contribute to the Josephson current. The leading contribution is then of second order in *H*_int_,

[20]
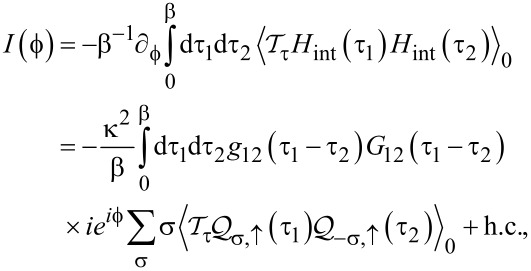


with 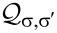 in Equation 13 and the small dimensionless parameter

[21]
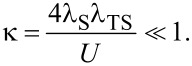


From Equation 7 and Equation 8, the bGF matrix elements needed in Equation 20 follow as

[22]
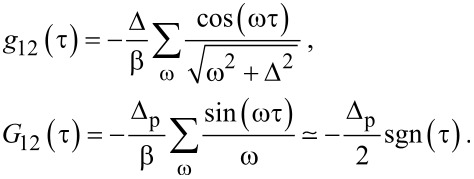


Now |*g*_12_(τ)| is exponentially small unless Δ|τ| *<* 1. In particular, *g*_12_(τ) → −δ(τ) for Δ → ∞. Moreover, for *B* 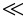 Δ with *B* ≡ |**B**|, the magnetic impurity (**S**) dynamics will be slow on time scales of the order of 1/Δ. We may therefore approximate the spin–spin correlators in Equation 20 by their respective equal-time expressions,

[23]
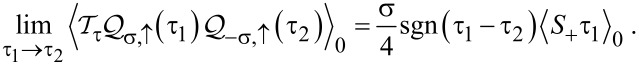


Inserting Equation 22 and Equation 23 into the expression for the supercurrent in Equation 20, the time integrations can be carried out analytically.

We obtain the CPR in the cotunneling regime as

[24]
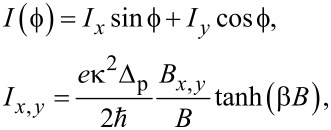


with κ in Equation 21. We note that while *I*(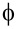) is formally independent of Δ, the value of Δ must be sufficiently large to justify the steps leading to Equation 24. Remarkably, Equation 24 predicts anomalous supercurrents for the S–QD–TS setup, i.e., a finite Josephson current for vanishing phase difference (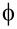 = 0) [45–46,72]. One can equivalently view this effect as a φ_0_-shift in the CPR, *I*(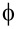) = *I_c_* sin(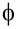 + φ_0_). An observation of this φ_0_-junction behavior could then provide additional evidence for MBSs (see also [47]), where Equation 24 shows that the local magnetic field is required to have a finite *B_y_*-component with 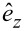 defining the MBS spin polarization direction. In particular, if **B** is aligned with 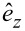, the supercurrent in Equation 24 vanishes identically since *s*-wave Cooper pairs cannot tunnel from the S lead into the TS wire in the absence of spin flips [31]. Otherwise, the CPR is 2π-periodic and sensitive to the MBS through the peculiar dependence on the relative orientation between the MBS spin polarization (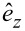) and the local Zeeman field **B** on the QD. The fact that *B_y_* ≠ 0 (rather than *B_x_* ≠ 0) is necessary to have φ_0_ ≠ 0 can be traced back to our choice of real-valued tunnel couplings. For tunable tunnel phases, also the field direction where one has φ_0_ = 0 will vary accordingly.

Noting that the anomalous Josephson effect has recently been observed in S–QD–S devices [73], we expect that similar experimental techniques will allow to access the CPR (Equation 24). We mention in passing that previous work has also pointed out that experiments employing QDs between N (instead of S) leads and TS wires can probe nonlocal effects due to MBSs [12,16,74–78]. In our case, e.g., by variation of the field direction in the *xy*-plane, Equation 24 predicts a tunable anomalous supercurrent. We conclude that in the cotunneling regime, the π-junction behavior of S–QD–S devices is replaced by the more exotic physics of φ_0_-junctions in the S–QD–TS setting.

#### Mean-field approximation

Next we present a mean-field analysis of the Hamiltonian (Equation 2) which allows us to go beyond the perturbative cotunneling regime. For the corresponding S–QD–S case, see [58,79]. We note that a full solution of this interacting many-body problem requires a detailed numerical analysis using, e.g., the numerical renormalization group [60–61] or quantum Monte Carlo simulations [59,63], which is beyond the scope of the present work. We start by defining the GF of the QD,

[25]
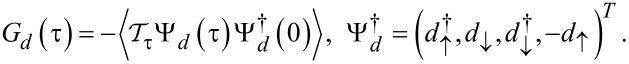


Note that this notation introduces double counting, which implies that only half of the levels are physically independent. Of course, the results below take this issue into account.

With the above Nambu bi-spinor basis, the mean-field Hamiltonian has the 4 × 4 matrix representation

[26]
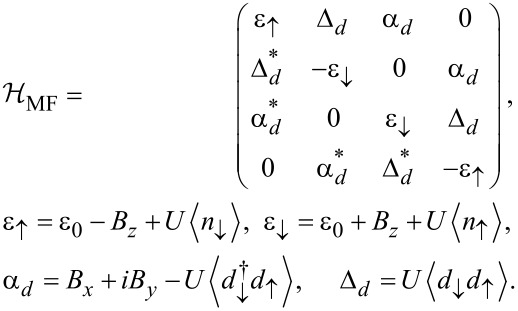


The mean-field parameters appearing in Equation 26 follow by solving the self-consistency equations

[27]
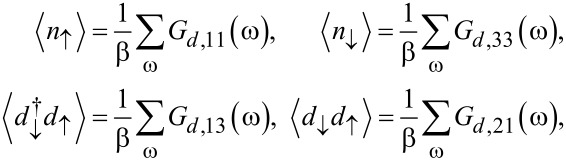


where the mean-field approximation readily yields

[28]
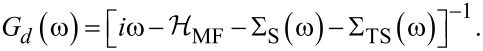


The self-energies Σ_S/TS_(ω) due to the coupling of the QD to the S/TS leads have the matrix representation

[29]
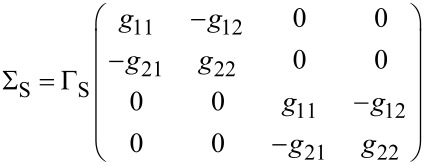


and

[30]
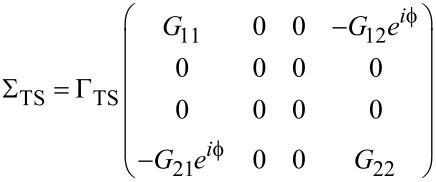


with the hybridization parameters Γ_S/TS_ = 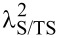. The bGFs *g*(ω) and *G*(ω) have been defined in Equation 7 and Equation 8, respectively. Once a self-consistent solution to Equation 27 has been determined, which in general requires numerics, the Josephson current is obtained from Equation 6 as

[31]
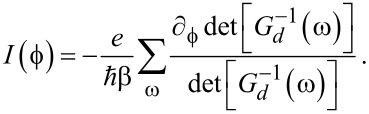


In what follows, we study a setup with Δ_p_ = Δ and consider the zero-temperature limit.

In order to compare our self-consistent mean-field results to the noninteracting case, let us briefly summarize analytical expressions for the *U* = 0 ABS spectrum in the atomic limit defined by Γ_S,TS_ 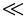 Δ. First we notice that at low energy scales, the self-energy Σ = Σ_S_ + Σ_TS_, see Equation 29 and Equation 30, simplifies to

[32]
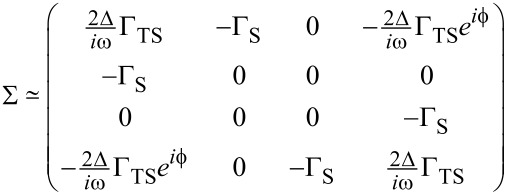


The ABS spectrum of the S–QD–TS junction then follows by solving a determinantal equation, 
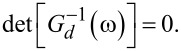
 One finds a zero-energy pole which is related to the MBS and results from the 1/ω dependence of Σ_TS_(ω). In addition, we get finite-energy subgap poles for

[33]
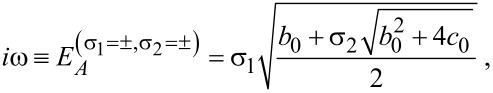


with the notation

[34]
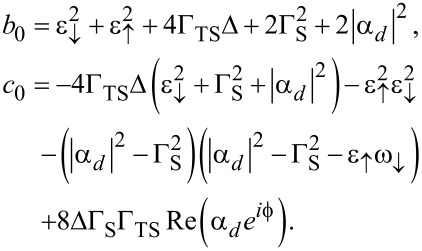


**Figure 2:**
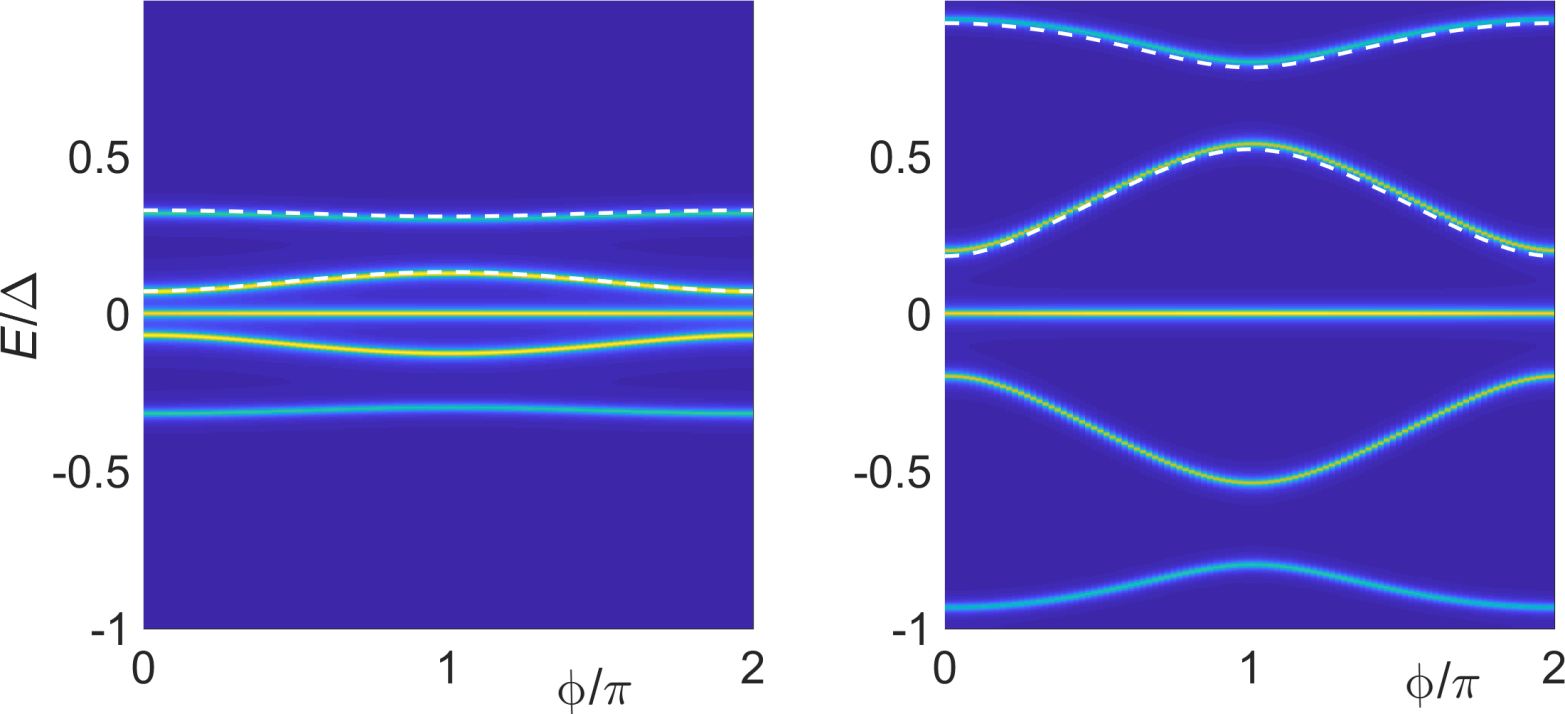
Phase dependence of the subgap spectrum of an S–QD–TS junction in the noninteracting case, *U* = 0. The TS wire is modeled from the low-energy limit of a Kitaev chain, and we use the parameters *B_y_* = 0, *B_x_* = *B_z_* = *B*/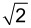, ε_0_ = 0, Δ_p_ = Δ, and Γ_S_ = Γ_TS_ = Γ. From blue to yellow, the color code indicates increasing values of the spectral density. The left (right) panel is for Γ = 0.045Δ and *B* = 0.1Δ (Γ = *B* = 0.5Δ). Solid curves were obtained by numerical evaluation of Equation 31. Dashed curves give the analytical prediction (Equation 33). In the right panel, the energies resulting from Equation 33 have been rescaled by the factor 1 + Γ/Δ.

In Figure 2, numerically exact results for the *U* = 0 ABS spectrum are compared to the analytical prediction (Equation 33). We first notice that, as expected, Equation 33 accurately fits the numerical results in the atomic limit, see the left panel in Figure 2. Deviations can be observed for larger values of Γ_S,TS_/Δ. However, as shown in the right panel of Figure 2, rather good agreement is again obtained by rescaling Equation 33 with a constant factor of the order of (1 + Γ_S,TS_/Δ). For finite *B_y_*, we find (data not shown) that the phase-dependent ABS spectrum is shifted with respect to 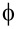 = 0. In fact, since the phase dependence of the subgap states comes from the term 
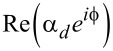
 in the atomic limit, see Equation 26 and Equation 34, *B_y_* can be fully accounted for in this limit by simply shifting 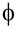 → 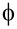 + φ_0_. We thereby recover the φ_0_-junction behavior discussed before for the cotunneling regime, see Equation 24.

**Figure 3:**
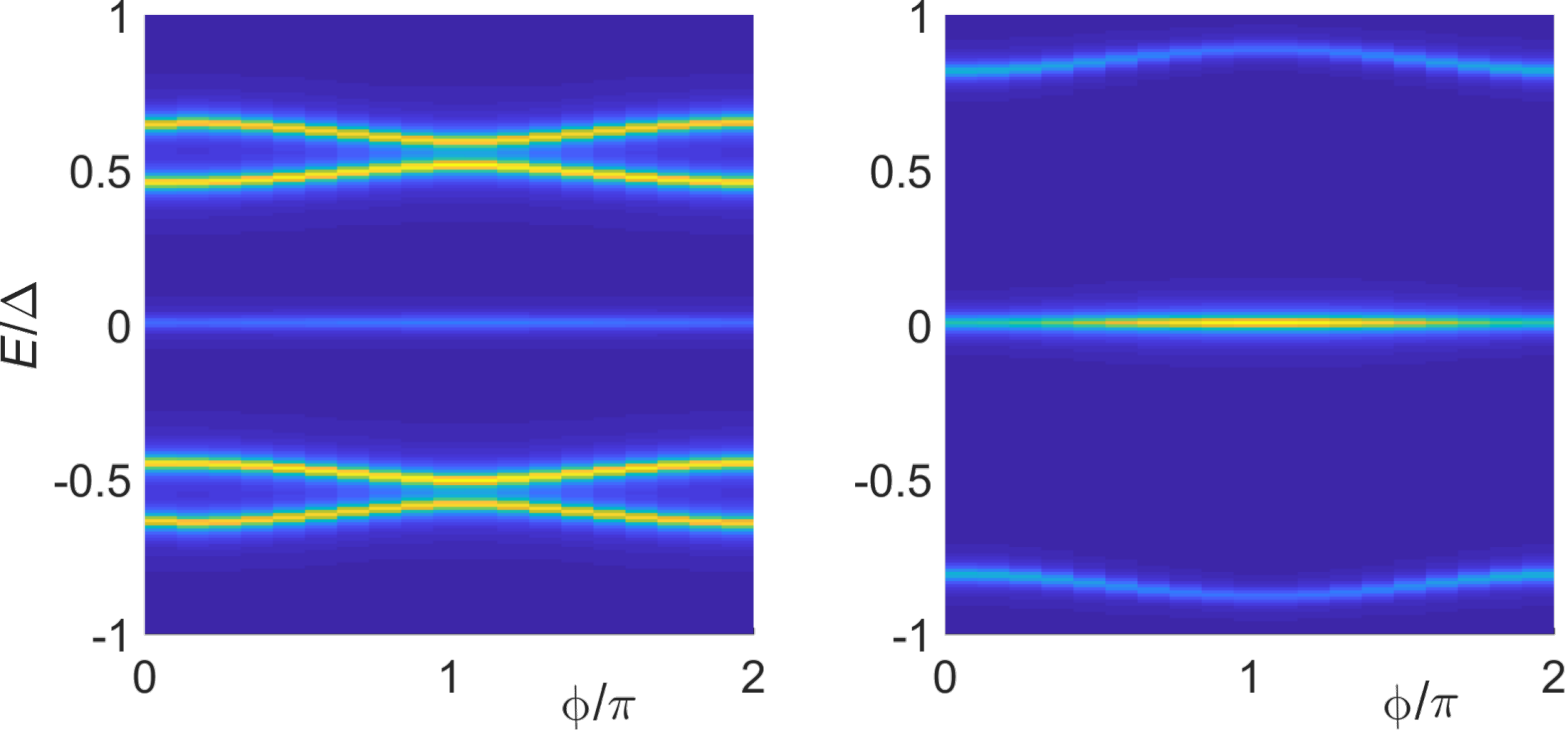
Phase-dependent ABS spectrum from mean-field theory for S–QD–TS junctions as in Figure 2 but with *U >* 0 and ε_0_ = −*U*/2. We put Δ_p_ = Δ, *B_y_* = 0, and Γ_S_ = Γ_TS_ = Γ. The color code is as in Figure 2. The left panel is for *U* = Δ, Γ = 0.5Δ, and *B_x_* = *B_z_* = *B*/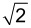 with *B* = 0.5Δ [cf. the right panel of Figure 2]. The right panel is for *U* = 10Δ, Γ = 4.5Δ, *B_x_* = 15Δ, and *B_z_* = 0.

We next turn to self-consistent mean-field results for the phase-dependent ABS spectrum at finite *U*. Figure 3 shows the spectrum for the electron–hole symmetric case ε_0_ = −*U*/2, with other parameters as in the right panel of Figure 2. For moderate interaction strength, e.g., taking *U* = Δ (left panel), we find that compared to the *U* = 0 case in Figure 2, interactions push together pairs of Andreev bands, e.g., the pair corresponding to 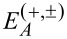 in Equation 31. On the other hand, for stronger interactions, e.g., *U* = 10Δ (right panel), the outer ABSs leak into the continuum spectrum and only the inner Andreev states remain inside the superconducting gap. The ABS spectrum shown in Figure 3 is similar to what is observed in mean-field calculations for S–QD–S systems with broken spin symmetry and in the magnetic regime of the QD, where one finds up to four ABSs for *U <* Δ while the outer ABSs merge with the continuum for *U >* Δ [79]. Interestingly, the inner ABS contribution to the free energy for *U* = 10Δ is minimal for 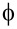 = π, see right panel of Figure 3, and we therefore expect π-junction behavior for *B_y_* = 0 also in the regime with *U* 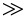 Δ and *B* 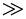 Δ. We notice, however, that changing the sign of *B_x_* would result in zero junction behavior. We interpret the inner ABSs for *U* 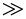 Δ as Shiba states with the phase dependence generated by the coupling to the MBS. Without the latter coupling, the Shiba state has 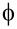-independent energy slightly below Δ determined by the scattering phase shift difference between both spin polarizations [80].

**Figure 4:**
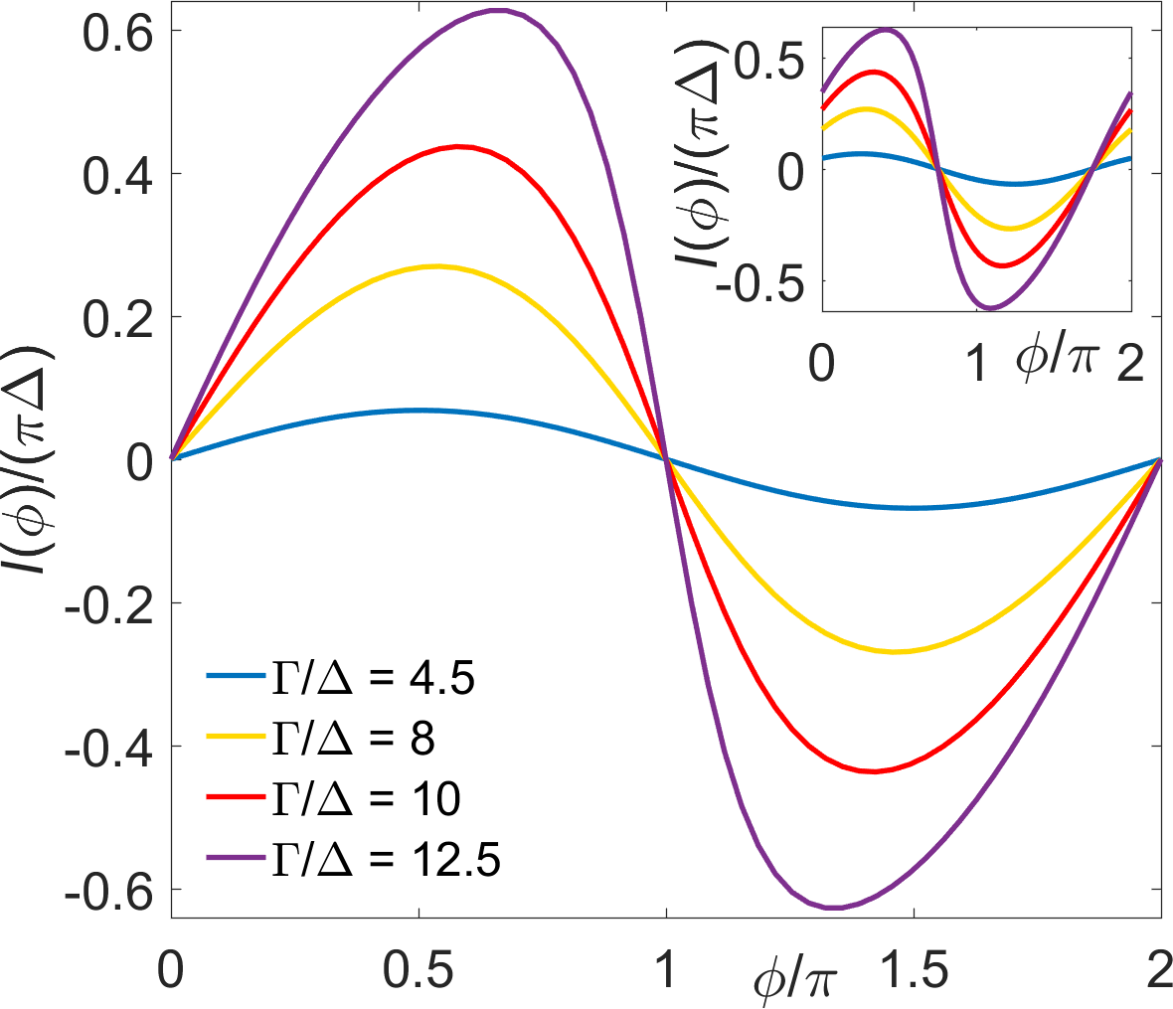
Main panel: Mean-field results for the CPR of S–QD–TS junctions with different Γ/Δ values, where we assume Δ*_p_* = Δ, *U* = 10Δ, ε_0_ = −*U*/2, Γ*_S_* = Γ_TS_ = Γ, *B* = 15Δ, and *B_z_* = 0. Main panel: For *B_x_* = −*B* and *B_y_* = 0. Inset: Same but for *B_y_* = −*B_x_* = *B*/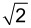, where φ_0_-junction behavior occurs.

As illustrated in Figure 4, the CPR computed numerically from Equation 31 for different values of Γ_S,TS_/Δ, where *B_x_* has been inverted with respect to its value in Figure 3, results in zero junction behavior. This behavior is expected from Equation 24 in the cotunneling regime, and Figure 4 shows that it also persists for Γ_S,TS_ 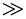 Δ. In contrast to Equation 24, however, the CPR for Γ_S,TS_ 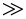 Δ differs from a purely sinusoidal behavior, see Figure 4. Moreover, for *B_y_* ≠ 0, we again encounter φ_0_-junction behavior, cf. the inset of Figure 4, in accordance with the perturbative result in Equation 24. Our mean-field results suggest that φ_0_-junction behavior is very robust and extends also into other parameter regimes as long as the condition *B_y_* ≠ 0 is met.

**Figure 5:**
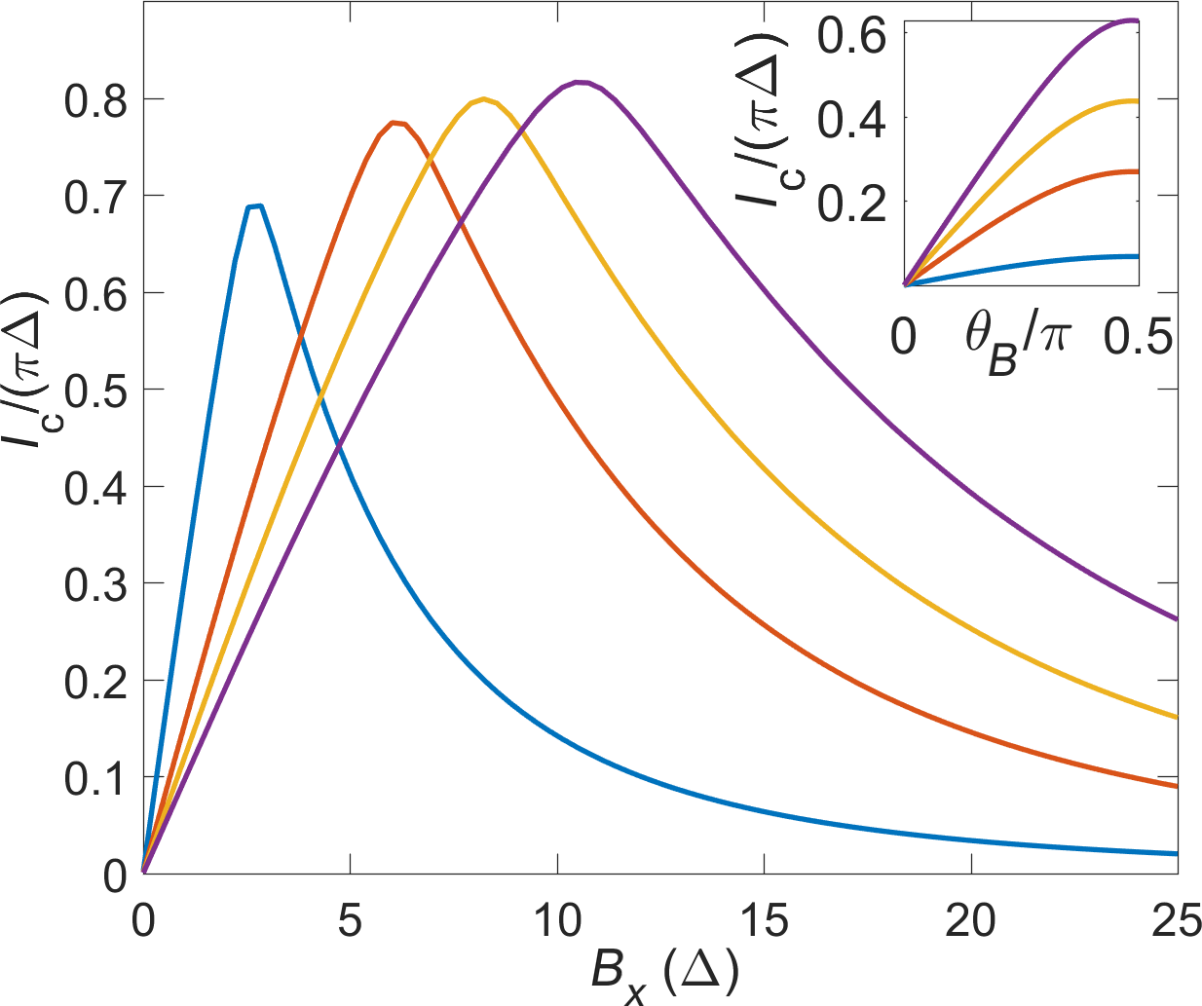
Main panel: Mean-field results for the critical current *I*_c_ vs local magnetic field scale *B_x_* in S–QD–TS junctions. Parameters are as in the main panel of Figure 4, i.e., *U* = 10Δ, ε_0_ = −*U*/2, and *B*_y,z_ = 0. From left to right, different curves are for Γ/Δ = 4.5, 8, 10 and 12.5. Inset: *I*_c_ vs angle θ*_B_*, where **B** = *B* (sinθ*_B_*,0,cosθ*_B_*) with *B* = 15Δ.

Next, Figure 5 shows mean-field results for the critical current, 
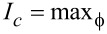
|*I*(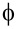)|, as function of the local magnetic field *B_x_* and otherwise the same parameters as in Figure 4. The main panel in Figure 5 shows that *I*_c_ increases linearly with *B_x_* for small *B_x_* *<* Δ, then exhibits a maximum around *B_x_* ≈ Γ, and subsequently decreases again to small values for *B_x_* 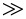 max{Γ_S,TS_,Δ}. On the other hand, for a fixed absolute value *B* of the magnetic field and *B_y_* = 0, the critical current also exhibits a maximum as a function of the angle θ*_B_* between **B** and the MBS spin polarization axis (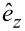). This effect is illustrated in the inset of Figure 5. As expected, the Josephson current vanishes for θ*_B_* → 0, where the supercurrent blockade argument of [31] implies *I*_c_ = 0, and reaches its maximal value for θ*_B_* = π/2.

### Spinful nanowire model for the TS

#### Model

Before turning to the S–TS–S setup in Figure 1b, we address the question of how the above results for S–QD–TS junctions change when using the spinful nanowire model of [2–3] instead of the low-energy limit of a Kitaev chain, see Equation 8. In fact, we will first describe the Josephson current for the elementary case of an S–TS junction using the spinful nanowire model. Surprisingly, to the best of our knowledge, this case has not yet been addressed in the literature.

In spatially discretized form, the spinful nanowire model for TS wires reads [2–3,43]

[35]
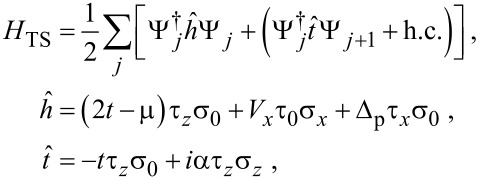


where the lattice fermion operators *c_jσ_* for given site *j* with spin polarizations σ = ↑,↓ are combined to the four-spinor operator


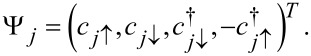


The Pauli matrices τ*_x,y,z_* (and unity τ_0_) again act in Nambu space, while Pauli matrices σ*_x,y,z_* and σ_0_ refer to spin. In the figures shown below, we choose the model parameters in Equation 35 as discussed in [43]. The lattice spacing is set to *a* = 10 nm, which results in a nearest-neighbor hopping *t* = 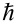
^2^/(2m*a^2^) = 20 meV and the spin–orbit coupling strength α = 4 meV for InAs nanowires. The proximity-induced pairing gap is again denoted by Δ_p_, the chemical potential is μ, and the bulk Zeeman energy scale *V_x_* is determined by a magnetic field applied along the wire. Under the condition

[36]
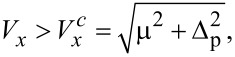


the topologically nontrivial phase is realized [2–3]. As we discuss below, the physics of the S–QD–TS junction sensitively depends on both the bulk Zeeman field *V_x_* and on the local magnetic field **B** acting on the QD, where one can either identify both magnetic fields or treat **B** as independent field. In any case, the bGF 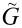
(ω) for the model in Equation 35, which now replaces the Kitaev chain result *G*(ω) in Equation 8, needs to be computed numerically. The bGF 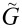 has been described in detail in [43], where also a straightforward numerical scheme for calculating 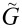
(ω) has been devised. With the replacement *G*→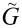, we can then take over the expressions for the Josephson current discussed before. Below we study these expressions in the zero-temperature limit.

#### S–TS junction

Let us first address the CPR for the S–TS junction case. The Josephson current can be computed using the bGF expression for tunnel junctions in [40], which is a simplified version of the above expressions for the S–QD–TS case. The spin-conserving tunnel coupling λ defines a transmission probability (transparency) 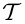 of the normal junction [40,43]. Close to the topological transition, the transparency is well approximated by

[37]
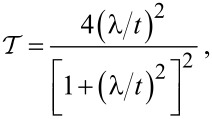


where *t* = 20 meV is the hopping parameter in Equation 35. We then study the CPR and the resulting critical current *I*_c_ as a function of 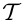 for both the topologically trivial (*V_x_* *<* 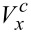) and the nontrivial (*V_x_* *>* 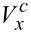) regime, see Equation 36.

**Figure 6:**
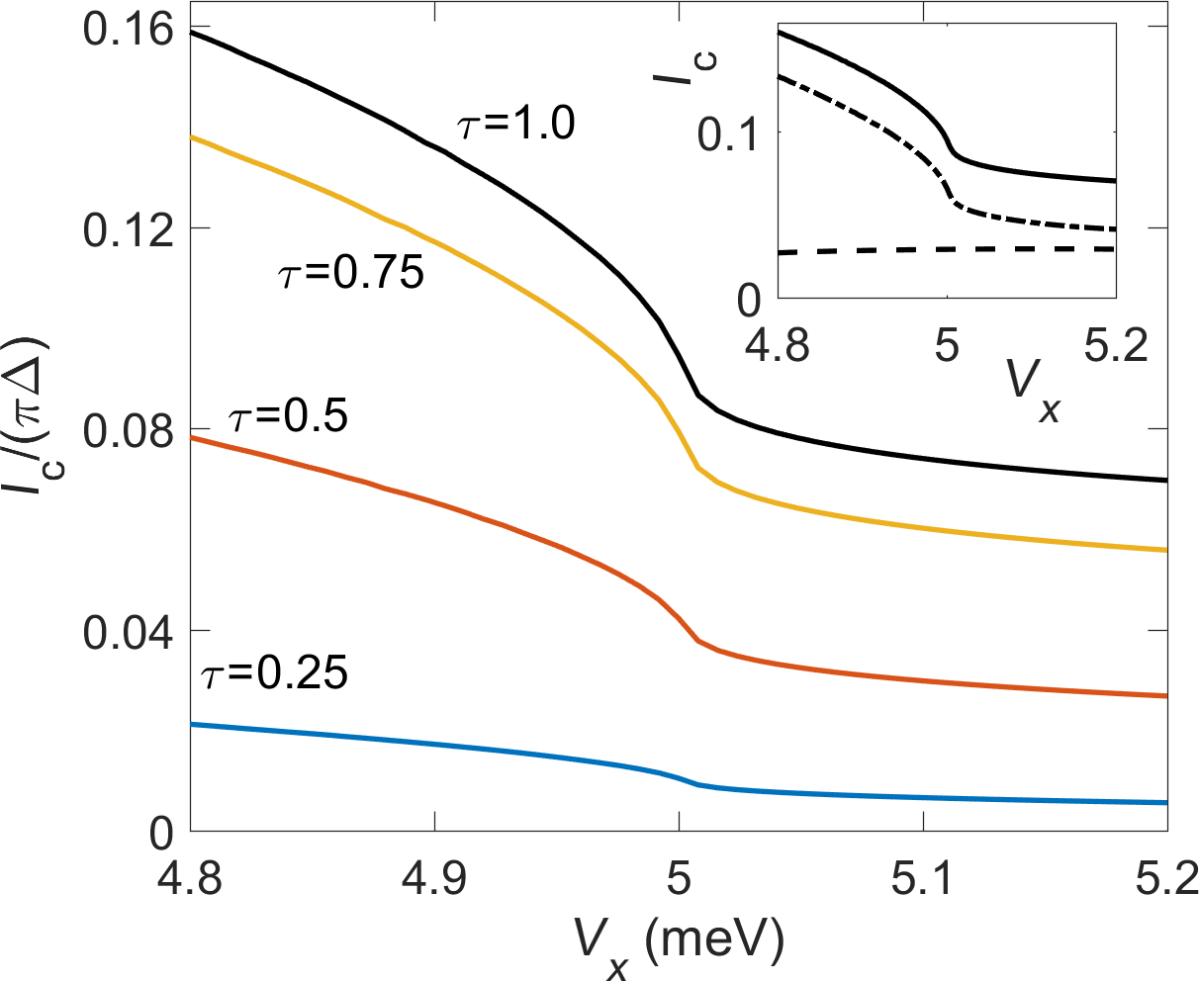
Main panel: Critical current *I*_c_ vs Zeeman energy *V_x_* for an S–TS junction using the spinful TS nanowire model (Equation 35) for Δ_p_ = Δ = 0.2 meV, μ = 5 meV, and different transparencies 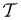 calculated from Equation 37. All other parameters are specified in the main text. Inset: Decomposition of *I*_c_ for 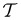 = 1 into ABS (dotted-dashed) and continuum (dashed) contributions.

In Figure 6, we show the *V_x_* dependence of the critical current *I*_c_ for the symmetric case Δ = Δ_p_. In particular, it is of interest to determine how *I*_c_ changes as one moves through the phase transition in Equation 36. First, we observe that *I*_c_ is strongly suppressed in the topological phase in comparison to the topologically trivial phase. In fact, *I*_c_ slowly decreases as one moves into the deep topological phase by increasing *V_x_*. This observation is in accordance with the expected supercurrent blockade in the deep topological limit [31]: *I*_c_ = 0 for the corresponding Kitaev chain case since *p*-wave pairing correlations on the TS side are incompatible with *s*-wave correlations on the S side. However, a residual finite supercurrent can be observed even for rather large values of *V_x_*. We attribute this effect to the remaining *s*-wave pairing correlations contained in the spinful nanowire model (Equation 35). Second, Figure 6 shows kink-like features in the *I*_c_(*V_x_*) curve near the topological transition, *V_x_* ≈ 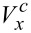. The inset of Figure 6 demonstrates that this feature comes from a rapid decrease of the ABS contribution while the continuum contribution remains smooth. This observation suggests that continuum contributions in this setup mainly originate from *s*-wave pairing correlations which are not particularly sensitive to the topological transition.

**Figure 7:**
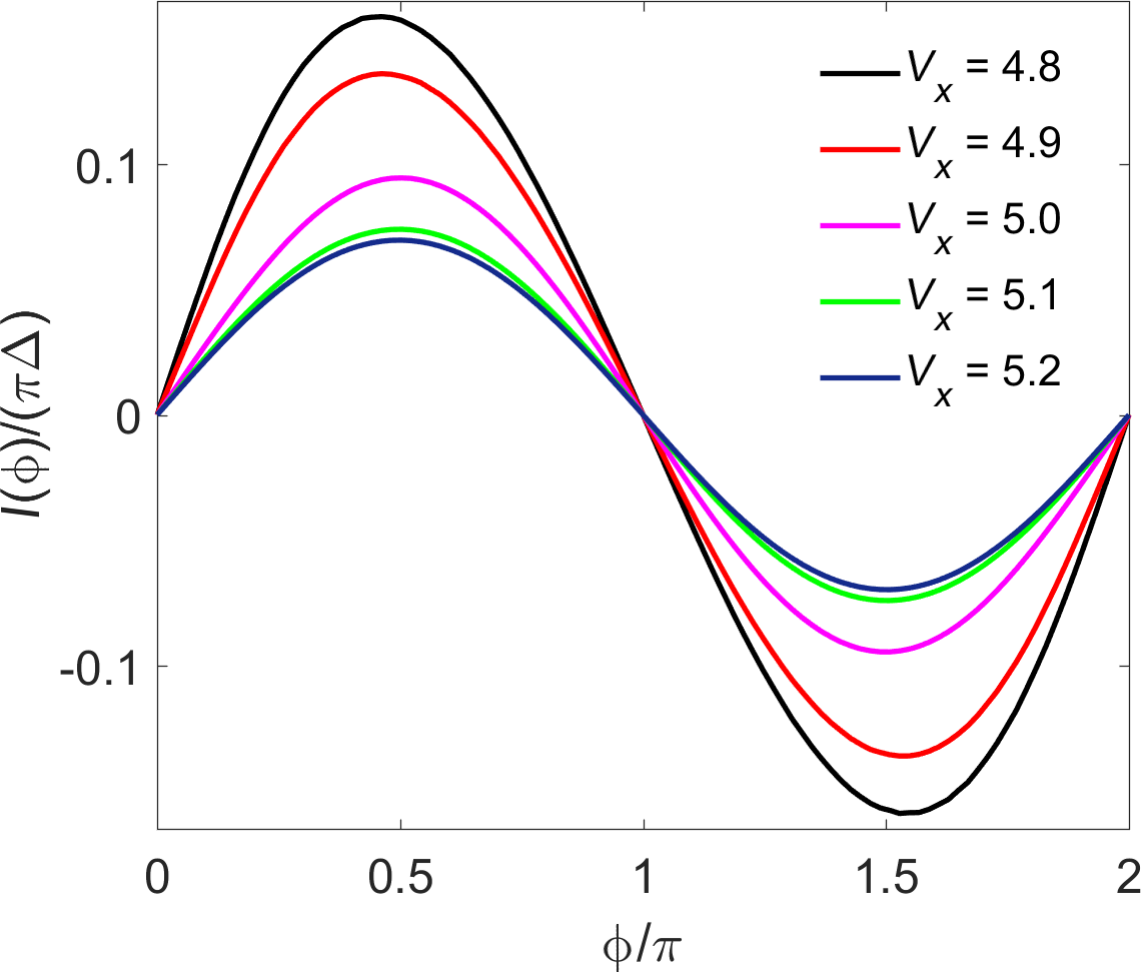
CPR for the S–TS junction with 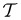 = 1 in Figure 6, for different bulk Zeeman fields *V_x_* (in meV) near the critical value 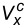 = 5.004 meV.

In Figure 7, we show the CPR for the S–TS junction with 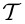 = 1 in Figure 6, where different curves correspond to different Zeeman couplings *V_x_* near the critical value. We find that in many parameter regions, in particular for 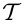 *<* 1, the CPR is to high accuracy given by a conventional 2π-periodic Josephson relation, *I*(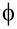) = *I*_c_ sin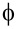. In the topologically trivial phase, small deviations from the sinusoidal law can be detected, but once one enters the topological phase, these deviations become extremely small.

#### S–QD–TS junction with spinful TS wire: Mean-field theory

Apart from providing a direct link to experimental control parameters, another advantage of using the spinful nanowire model of [2–3] for modeling the TS wire is that the angle between the local Zeeman field **B** and the MBS spin polarization does not have to be introduced as phenomenological parameter but instead results from the calculation [43]. It is thus interesting to study the Josephson current in S–QD–TS junctions where the TS wire is described by the spinful nanowire model. For this purpose, we now revisit the mean-field scheme for S–QD–TS junctions using the bGF 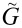
(ω) for the spinful nanowire model (Equation 35). In particular, with the replacement *G*→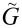, we solve the self-consistency equations (Equation 27) and thereby obtain the mean-field parameters in Equation 26. The resulting QD GF, *G_d_*(ω) in Equation 28, then determines the Josephson current in Equation 31. Below we present self-consistent mean-field results obtained from this scheme. In view of the huge parameter space of this problem, we here only discuss a few key observations. A full discussion of the phase diagram and the corresponding physics will be given elsewhere.

**Figure 8:**
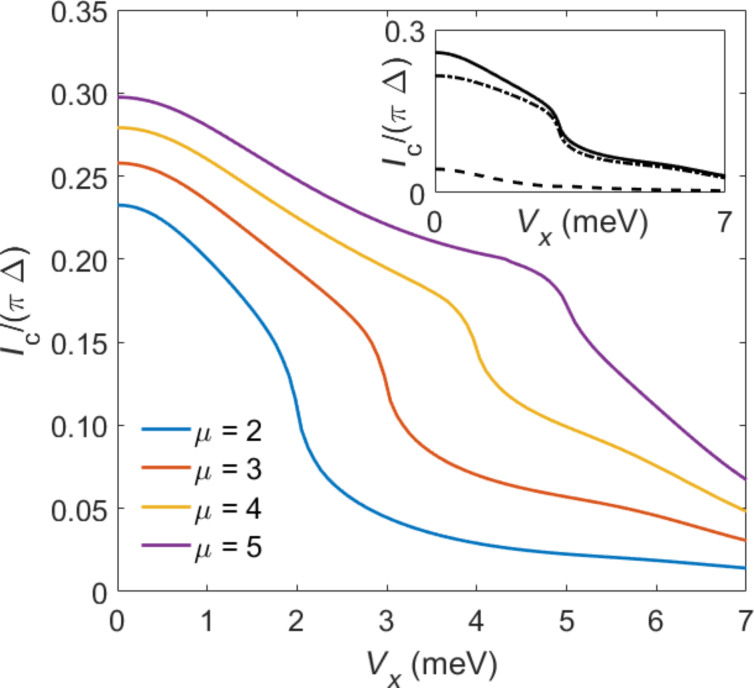
Main panel: Critical current *I*_c_ vs Zeeman energy *V_x_* for S–QD–TS junctions from mean-field theory using the spinful TS nanowire model (Equation 35). Results are shown for several values of the chemical potential μ (in meV), where we assume *U* = 10Δ, ε_0_ = −*U*/2, Δ_p_ = Δ = 0.2 meV, Γ*_S_* = 2Γ_TS_ = 9Δ, and **B** = (*V_x_*,0,0). Inset: Detailed view of the transition region *V_x_* ≈ 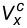 for μ = 4 meV, including a decomposition of *I*_c_ into the ABS (dotted-dashed) and the continuum (dashed) contribution.

The main panel of Figure 8 shows the critical current *I*_c_ vs the bulk Zeeman energy *V_x_* for several values of the chemical potential μ, where the respective critical value 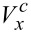 in Equation 36 for the topological phase transition also changes with μ. The results in Figure 8 assume that the local magnetic field **B** acting on the QD coincides with the bulk Zeeman field *V_x_* in the TS wire, i.e., **B** = (*V_x_*,0,0). For the rather large values of Γ_S,TS_ taken in Figure 8, the *I*_c_ vs *V_x_* curves again exhibit a kink-like feature near the topological transition, *V_x_* ≈ 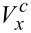. This behavior is very similar to what happens in S–TS junctions with large transparency 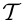, cf. Figure 6. As demonstrated in the inset of Figure 8, the physical reason for the kink feature can be traced back to a sudden drop of the ABS contribution to *I*_c_ when entering the topological phase *V_x_* *>* 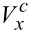. In the latter phase, *I*_c_ becomes strongly suppressed in close analogy to the S–TS junction case shown in Figure 6.

**Figure 9:**
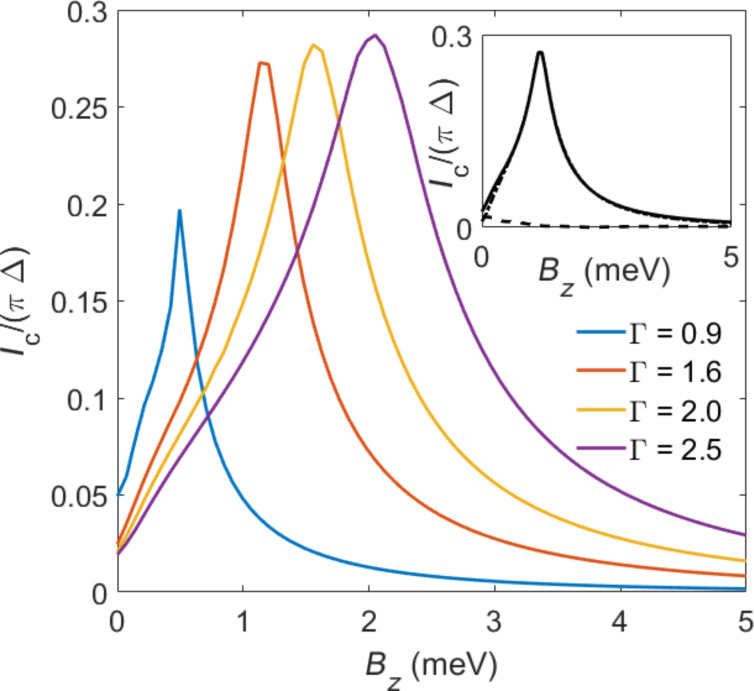
Main panel: Mean-field results for *I*_c_ vs *B_z_* in S–QD–TS junctions for several values of Γ*_S_* = Γ_TS_ = Γ (in meV) and μ = 4 meV. The bulk Zeeman field *V_x_* = 5 meV along 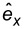 (where *V_x_* *>* 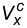 for our parameters) is applied to the spinful TS wire, while the QD is subject to the local magnetic field **B** = *B_z_*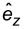. All other parameters are as in Figure 8. Inset: Decomposition of *I*_c_ into ABS (dotted-dashed) and continuum (dashed) contributions for Γ = 1.6 meV.

In Figure 8, both the QD and the TS wire were subject to the same magnetic Zeeman field. If the direction and/or the size of the local magnetic field **B** applied to the QD can be varied independently from the bulk magnetic field *V_x_*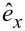 applied to the TS wire, one can arrive at rather different conclusions. To illustrate this statement, Figure 9 shows the *I*_c_ vs *B_z_* dependence for **B** = (0,0,*B_z_*) perpendicular to the bulk field, with *V_x_* *>* 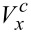 such that the TS wire is in the topological phase. In this case, Figure 9 shows that *I*_c_ exhibits a maximum close to *B_z_* ~ Γ. This behavior is reminiscent of what we observed above in Figure 5, using the low-energy limit of a Kitaev chain for the bGF of the TS wire. Remarkably, the critical current can here reach values close to the unitary limit, *I*_c_ *~ e*Δ/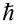. We note that since *B_z_* does not drive a phase transition, no kink-like features appear for the *I*_c_(*B_z_*) curves shown in Figure 9. Finally, the inset of Figure 9 shows that for **B** perpendicular to *V_x_*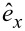, where *V_x_* *>* 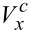 for the parameters chosen in Figure 9, the ABSs provide the dominant contribution to the current in this regime.

### S–TS–S junctions: Switching the parity of a superconducting atomic contact

#### Model

We now proceed to the three-terminal S–TS–S setup shown in Figure 1b. The CPR found in the related TS–S–TS trijunction case has been discussed in detail in [43], see also [44]. Among other findings, a main conclusion of [43] for the TS–S–TS geometry was that the CPR can reveal information about the spin canting angle between the MBS spin polarization axes in both TS wires. In what follows, we study the superficially similar yet rather different case of an S–TS–S junction. Throughout this section, we model the TS wire via the low-energy theory of a spinless Kitaev chain, where the bGF *G*(ω) in Equation 8 applies.

One can view the setup in Figure 1b as a conventional superconducting atomic contact (SAC) with a TS wire tunnel-coupled to the S–S junction. Over the past few years, impressive experimental progress [52–54] has demonstrated that the ABS level system in a SAC [81] can be accurately probed and manipulated by coherent or incoherent microwave spectroscopy techniques. We show below that an additional TS wire, cf. Figure 1b, acts as tunable parity switch on the many-body ABS levels of the SAC. As we have discussed above, the supercurrent flowing directly between a given S lead and the TS wire is expected to be strongly suppressed. However, through the hybridization with the MBS, Andreev level configurations with even and odd fermion parity are connected. This effect has profound and potentially useful consequences for Andreev spectroscopy.

An alternative view of the setup in Figure 1b is to imagine an S–TS junction, where S1 plays the role of the S lead and the spinful TS wire is effectively composed from a spinless (Kitaev) TS wire and the S2 superconductor. The *p*- and *s*-wave pairing correlations in the spinful TS wire are thereby spatially separated. Since the *s*- and *p*-wave bands represent normal modes, they are not directly coupled to each other in this scenario, i.e., we have to put λ_2_ = 0. We discuss this analogy in more detail later on.

We consider a conventional single-channel SAC (gap Δ) coupled via a point contact to a TS wire (gap Δ_p_), cf. Figure 1b. The superconducting phase difference across the SAC is denoted by 
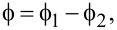
 where 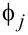 is the phase difference between the respective S arm (*j* = 1,2) and the TS wire. In practice, the SAC can be embedded into a superconducting ring for magnetic flux tuning of 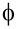. To allow for analytical progress, we here assume that Δ_p_ is so large that continuum quasiparticle excitations in the TS wire can be neglected. In that case, only the MBS at the junction has to be kept when modeling the TS wire. However, we will also hint at how one can treat the general case.

For the two S leads, boundary fermion fields are contained in Nambu spinors as in Equation 7,

[38]
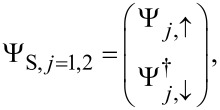


where their bGF follows with the Nambu matrix *g*(ω) in Equation 7 as

[39]
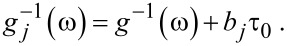


We again use Pauli matrices τ*_x,y,z_* and unity τ_0_ in Nambu space. The dimensionless parameters *b_1,2_* describe the Zeeman field component along the MBS spin polarization axis, see below. Since above-gap quasiparticles in the TS wire are neglected here, the TS wire is represented by the Majorana operator γ = γ^†^, with γ^2^ = 1/2, which anticommutes with all other fermions. We may represent γ by an auxiliary fermion *f*_↑_, where the index reminds us that the MBS spin polarization points along 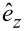,

[40]
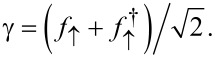


The other Majorana mode γ′ = 
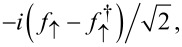
 which is localized at the opposite end of the TS wire, is assumed to have negligible hybridization with the Ψ_S_*_,j_* spinors and with γ. Writing the Euclidean action as *S* = *S*_0_ + *S*_tun_, we have an uncoupled action contribution,

[41]
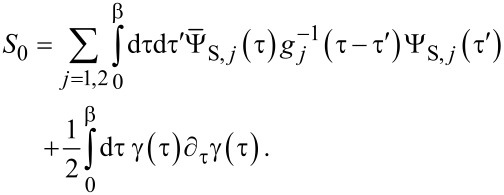


The leads are connected by a time-local tunnel action corresponding to the tunnel Hamiltonian

[42]
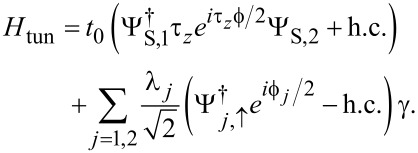


Without loss of generality, we assume that the tunnel amplitudes *t*_0_ and λ_1,2_, see Figure 1b, are real-valued and that they include density-of-state factors again. The parameter *t*_0_ (with 0 ≤ *t*_0_ ≤ 1) determines the transparency 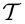 of the SAC in the normal-conducting state [36], cf. Equation 37,

[1]
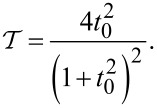


Note that in Equation 42 we have again assumed spin-conserving tunneling, where only spin-↑ fermions in the SAC are tunnel-coupled to the Majorana fermion γ, cf. Equation 5.

At this stage, it is convenient to trace out the Ψ_S,2_ spinor field. As a result, the SAC is described in terms of only one spinor field, Ψ ≡ Ψ_S,1_, which however is still coupled to the Majorana field γ. After some algebra, we obtain the effective action

[43]
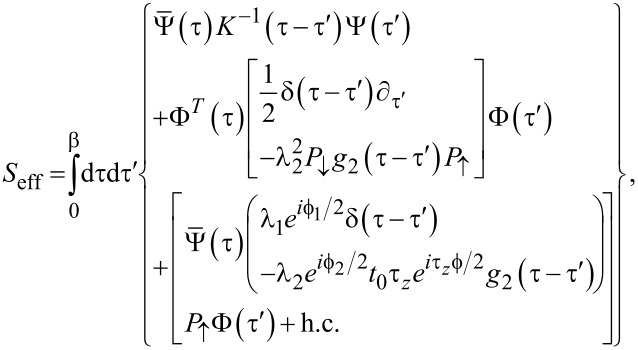


where the operator *P*_↑_ = (τ_0_ + τ*_z_*)/2 projects a Nambu spinor to its spin-↑ component. Moreover, we have defined an effective GF in Nambu space with frequency components

[44]
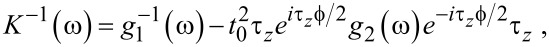


and the TS lead has been represented by the Majorana–Nambu spinor

[45]
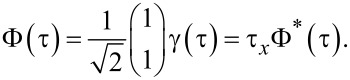


We note in passing that Equation 43 could at this point be generalized to include continuum states in the TS wire. To that end, one has to (i) replace Φ → (ψ, ψ^†^)*^T^*, where ψ is the boundary fermion of the effectively spinless TS wire, and (ii) replace δ(τ − τ′)∂_τ′_ → *G*^−1^(τ − τ′) with *G* in Equation 8. Including bulk TS quasiparticles becomes necessary for small values of the proximity gap, Δ*_p_* 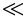 Δ, and/or when studying nonequilibrium applications within a Keldysh version of our formalism.

In any case, after neglecting the above-gap TS continuum quasiparticles, the partition function follows with *S*_eff_ in Equation 43 in the functional integral representation

[46]
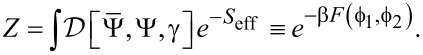


As before, the Josephson current through S lead no. *j* then follows from the free energy via


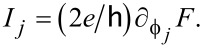


The supercurrent flowing through the TS wire is then given by

[47]
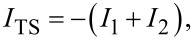


as dictated by current conservation.

#### Atomic limit

In order to get insight into the basic physics, we now analyze in detail the atomic limit, where Δ represents the largest energy scale of interest and hence the dynamics is confined to the subgap region. In this case, we can approximate 
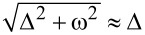
. After the rescaling


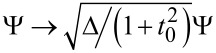


in Equation 43, we arrive at an effective action, *S*_eff_ → *S*_at_, valid in the atomic limit,

[48]
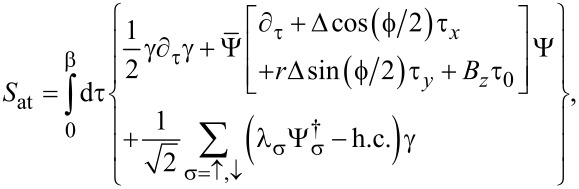


where 
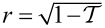
 is the reflection amplitude of the SAC, see Equation 1. We recall that 
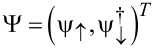
, see Equation 38. Moreover, we define the auxiliary parameters

[49]
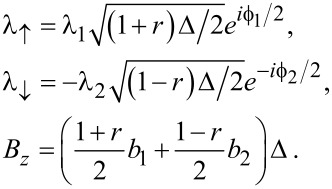


The parameters *b*_1,2_ in Equation 39 thus effectively generate the Zeeman scale *B_z_* in Equation 49.

As a consequence of the atomic limit approximation, the action *S*_at_ in Equation 48 is equivalently expressed in terms of the effective Hamiltonian

[50]
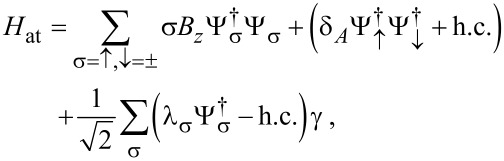


where we define

[51]
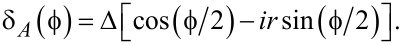


For a SAC decoupled from the TS wire and taken at zero field (*B_z_* = 0), the ABS energy follows from Equation 50 in the standard form [62]

[52]
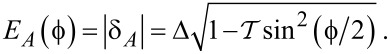


We emphasize that *H*_at_ neglects TS continuum quasiparticles as well as all types of quasiparticle poisoning processes. Let us briefly pause in order to make two remarks. First, we note that the Majorana field


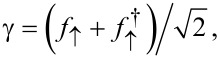


see Equation 40, couples to both spin modes ψ_σ_ in Equation 50. The coupling λ_↓_ between γ and the spin-↓ field in the SAC, ψ_↓_, is generated by crossed Andreev reflection processes, where a Cooper pair in lead S2 splits according to 
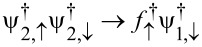
, plus the conjugate process. Second, we observe that *H*_at_ is invariant under a particle–hole transformation, amounting to the replacements 
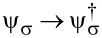
 and 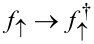, along with *B_z_* → − *B_z_* and 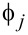 → 2π − 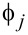.

We next notice that with *n*_σ_ = 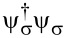 = 0,1 and *n_f_* = 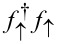 = 0,1, the total fermion parity of the junction,

[53]
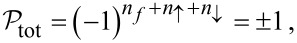


is a conserved quantity, [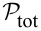, *H*_at_]_−_ = 0. Below we restrict our analysis to the even-parity sector 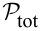 = +1, but analogous results hold for the odd-parity case. The corresponding Hilbert subspace is spanned by four states,

[54]
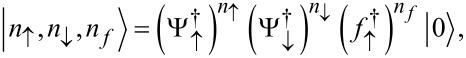


where (*n*_↑_, *n*_↓_, *n_f_*) 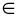 {(0,0,0), (1,1,0), (1,0,1), (0,1,1)} and 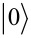 is the vacuum state. In this basis, the Hamiltonian (Equation 50) has the matrix representation

[55]
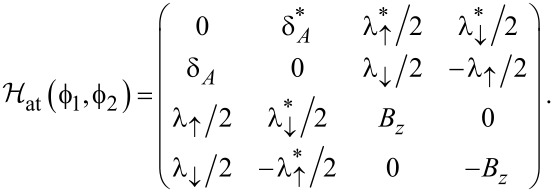


The even-parity ground state energy, 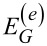 = min(ε), follows as the smallest root of the quartic equation

[56]
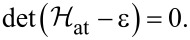


In order to obtain simple results, let us now consider the special case λ_2_ = 0, where the TS wire is directly coupled to lead S1 only, see Figure 1b. In that case, we also have λ_↓_= 0, see Equation 49, and Equation 56 implies the four eigenenergies ±ε_±_ with

[57]
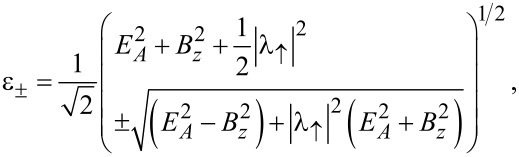


with 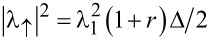, see Equation 49. The ground-state energy is thus given by 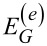 = −ε_+_. Since *E_G_* depends on the phases 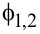 only via the Andreev level energy *E_A_*(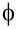) in Equation 52, the Josephson current through the SAC is given by

[58]
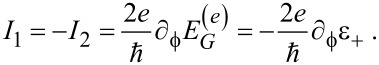


Note that Equation 47 then implies that no supercurrent flows into the TS wire.

Next we observe that in the absence of the TS probe (λ_1_ = 0), the even and odd fermion parity sectors of the SAC, 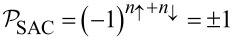, are decoupled, see Equation 55, and Equation 57 yields 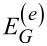 = −max(*E_A_*, |*B_z_*|). Importantly, the Josephson current is therefore fully blocked if the ground state is in the 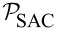 = −1 sector, i.e., for |*B_z_*| *> E_A_*(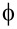). For λ_1_ ≠ 0, however, 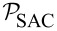 is not conserved anymore. This implies that the MBS can act as parity switch between the two Andreev sectors with parity 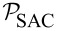 = ±1. Near the level crossing point at *E_A_* ≈ |*B_z_*|, i.e., assuming 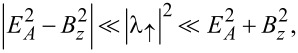 we obtain

[59]
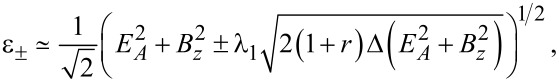


which implies a nonvanishing supercurrent through the SAC even in the field-dominated regime, |*B_z_*| *> E_A_*. The MBS therefore acts as a parity switch and leaves a trace in the CPR by lifting the supercurrent blockade.

#### Another interpretation

Interestingly, for λ_2_ = 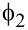 = 0, the S–TS–S setup in Figure 1b could also be viewed as a toy model for an S–TS junction, where the TS part corresponds to a spinful model. In that analogy, the Nambu spinor Ψ_S,1_ stands for the S lead while the spinful TS wire is represented by (i) the Nambu spinor Ψ_S,2_ which is responsible for the residual *s*-wave pairing correlations, and (ii) by the MF γ (or, more generally, by the Kitaev-chain spinless boundary fermion ψ) which encodes *p*-wave pairing correlations. Moreover, *t*_0_ and λ_1_ should now be understood as spin-conserving phenomenological tunnel couplings acting in the *s*–*s* and *s*–*p* wave channels, respectively. The phase difference across this effective S–TS junction is 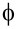 = 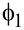 and the net S–TS tunnel coupling is given by 
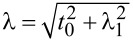
. Putting λ_1_ = 0 in the topologically trivial phase of the TS wire, the Josephson current carried by Andreev states in the *s*–*s* channel is blocked when the ground state is in the odd parity sector of the SAC. For λ_1_ ≠ 0, the MBS-mediated switching between odd and even parity sectors will now be activated and thereby lift the supercurrent blockade.

#### Conventional midgap level

A similar behavior as predicted above for the MBS-induced parity switch between 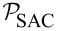 = ±1 sectors could also be expected from a conventional fermionic subgap state tunnel-coupled to the SAC. Such a subgap state may be represented, e.g., by a single-level quantum dot in the Coulomb blockade regime. In particular, for a midgap (zero-energy) level with the fermion operator *d*, the Hamiltonian *H*_at_ in Equation 50 has to be replaced with

[60]
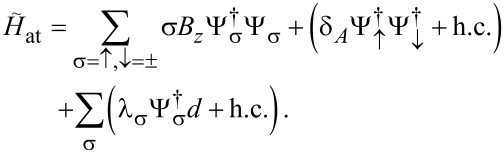


In the even total parity basis (Equation 54), the matrix representation of the Hamiltonian is then instead of Equation 55 given by

[61]
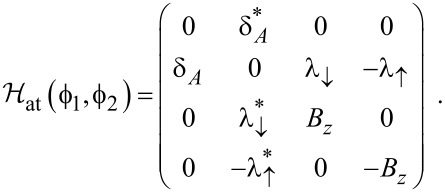


Assuming |λ_↑_| = |λ_↓_| ≡ λ, Equation 56 then yields the eigenenergies ±ε_±_ with

[62]
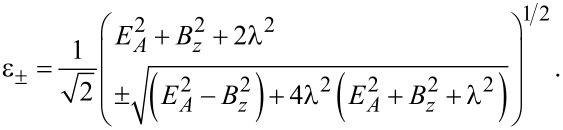


Remarkably, the ABS spectra in Equation 62 and Equation 57 are rather similar for 
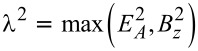
. However, the MBS will automatically be located at zero energy and thus represents a generic situation.

## Conclusion

We close this paper by summarizing our main findings. We have studied the Josephson effect in different setups involving both conventional *s*-wave BCS superconductors (S leads) and topologically nontrivial 1D *p*-wave superconductors (TS leads) with Majorana end states. The TS wires have been described either by a spinless theory applicable in the deep topological regime, which has the advantage of allowing for analytical progress but makes it difficult to establish contact to experimental control parameters, or by a spinful nanowire model as suggested in [2–3]. We have employed a unified imaginary-time Green’s function approach to analyze the equilibrium properties of such devices, but a Keldysh generalization is straightforward and allows one to study also nonequilibrium applications.

For S–TS tunnel junctions, we find that in the topological phase of the TS wire, the supercurrent is mainly carried by above-gap continuum contributions. We confirm the expected supercurrent blockade [31] in the deep topological regime (where the spinless theory is fully valid and thus no residual *s*-wave pairing exists), while for realistic parameters, a small but finite critical current is found. To good approximation, the Josephson current obeys the usual 2π-periodic sinusoidal current–phase relation. The dependence of the critical current on the bulk Zeeman field driving the TS wire through the topological phase transition shows a kink-like feature at the critical value, which is caused by a sudden drop of the Andreev state contribution.

The supercurrent blockade in the deep topological phase could be lifted by adding a magnetic impurity to the junction, also allowing for the presence of a local magnetic field **B**. Such a magnetic impurity arises from a spin-degenerate quantum dot (QD), and we have studied the corresponding S–QD–TS problem for both the spinless and the spinful TS wire model. Based on analytical results valid in the cotunneling regime as well as numerical results within the mean-field approximation, we predict φ_0_-junction behavior (anomalous Josephson effect) for the current–phase relation when the TS wire is in the topological phase.

As a final example for devices combining conventional and topological superconductors, we have shown that S–TS–S devices allow for a Majorana-induced parity switch between Andreev state sectors with different parity in a superconducting atomic contact. This observation could be useful for future microwave spectroscopy experiments of Andreev qubits in such contacts.
